# Temperature is a cryptic factor shaping the geographical pattern of genetic variation in *Ceratophyllum demersum* across a subtropical freshwater lake

**DOI:** 10.1016/j.pld.2023.08.002

**Published:** 2023-08-19

**Authors:** Yixian Li, Xuyao Zhao, Manli Xia, Xinzeng Wei, Hongwei Hou

**Affiliations:** a The State Key Laboratory of Freshwater Ecology and Biotechnology, The Key Laboratory of Aquatic Biodiversity and Conservation of Chinese Academy of Sciences, Institute of Hydrobiology, Chinese Academy of Sciences, Wuhan 430072, Hubei, China; b University of Chinese Academy of Sciences, Beijing 100049, China; c Key Laboratory of Aquatic Botany and Watershed Ecology, Wuhan Botanical Garden, Chinese Academy of Sciences, Wuhan 430074, Hubei, China

**Keywords:** Genetic diversity, Epigenetic variation, Temperature, Macrophyte, Restoration

## Abstract

Macrophyte habitats exhibit remarkable heterogeneity, encompassing the spatial variation of abiotic and biotic components such as changes in water conditions and weather as well as anthropogenic stressors. Environmental factors are thought to be important drivers shaping the genetic and epigenetic variation of aquatic plants. However, the links among genetic diversity, epigenetic variation, and environmental variables remain largely unclear, especially for clonal aquatic plants. Here, we performed population genetic and epigenetic analyses in conjunction with habitat discrimination to elucidate the environmental factors driving intraspecies genetic and epigenetic variation in hornwort (*Ceratophyllum demersum*) in a subtropical lake. Environmental factors were highly correlated with the genetic and epigenetic variation of *C. demersum*, with temperature being a key driver of the genetic variation. Lower temperature was detected to be correlated with greater genetic and epigenetic variation. Genetic and epigenetic variation were positively driven by water temperature, but were negatively affected by ambient air temperature. These findings indicate that the genetic and epigenetic variation of this clonal aquatic herb is not related to the geographic feature but is instead driven by environmental conditions, and demonstrate the effects of temperature on local genetic and epigenetic variation in aquatic systems.

## Highlights

• Genetic and epigenetic variation of *Ceratophyllum demersum* are analyzed under a temperature gradient.
• Two genetic clusters are existed among analyzed populations.
• Genetic and epigenetic variation of *C. demersum* are correlated with temperature.
• Higher temperature feature of habitat reveals lower genetic diversity and epigenetic variation in *C. demersum.*
• Species restoration should consider habitats situation when generate the property area.

## 1 Introduction

Environmental water conditions constantly drive the adaptation of aquatic plants to help them cope with dynamic variations in their habitats (Hedrick, 1978). During their adaptation to environmental shifts, plants undergo genetic and epigenetic changes to compensate for environmental oscillations (Robertson et al., 2017). The genetic and epigenetic diversity of plants in natural populations is generally related to environmental gradients (Richards et al., 2017). Clonal aquatic herbs species usually develop diverse phenotypic traits to adapt to the long-term effects of the environment (Wang et al., 2020). However, since epigenetic-induced phenotypic changes can be inherited through meiosis, epigenetic modifications represent more rapid responses to random environmental changes and anthropogenic stressors compared to genetic variation (Schulz et al., 2014).

Investigating the relative contributions of geographical and environmental variation to genetic divergence is critical for understanding adaptive differentiation during ecological speciation. Associations between environmental characteristics and the adaptive variations of species and their interactions have been widely investigated in various plants (Barajas-Barbosa et al., 2020; Ortego et al., 2012; Shen et al., 2022). Such studies have made considerable progress in understanding the relative roles of adaptive and nonadaptive processes in shaping the patterns of genomic variation and the effects of environmental variables on adaptive differentiation. Although researchers have recognized the importance of environmental variables to genetic structure and epigenetic variation, few studies have focused on aquatic plants, especially clonal macrophytes.

*Ceratophyllum demersum* L. (Ceratophyllaceae), commonly known as hornwort, is a submerged, rootless, free-floating aquatic macrophyte with cosmopolitan distribution. This species mainly reproduces vegetatively and occurs approximately 10 m offshore. *C. demersum* stems are up to 1–3 m long, and its branches can be modified as rhizoids (Cronk and Fennessy, 2016). *C. demersum* is sensitive to water pollution and has been established as a reference species to detect trace element pollution in freshwater ecosystems (Polechońska and Klink, 2021). Although *C. demersum* occurs widely in China and worldwide, where its environmental habitats are usually heterogeneous, previous studies have only characterized the differentiation of genotypes among geographical regions; the intraspecies variation and the associations between genetic and habitat variation remain uncharacterized (Hyldgaard et al., 2017). Meanwhile, since the implementation of a 10-years fishing ban in the Yangtze River in 2020, natural populations of *C. demersum* have been experiencing cycles of cascading growth and extirpation in a number of lakes, as observed during our field monitoring. Therefore, exploring how heterogeneous environments shape the genetic and epigenetic variation of *C. demersum* can provide useful information for the restoration of this species, not only in our study areas but also on a wider scale.

In this study, we investigated the levels of genetic and epigenetic variation in *Ceratophyllum* *demersum* across Liangzi Lake, the location with the highest level of macrophyte diversity in the Yangtze River basin, and compared the environmental components among habitats. Using amplified fragment polymorphism length (AFLP) and methylated-sensitive amplified polymorphism (MSAP) markers, we 1) investigated the genetic diversity and epigenetic variation of *C. demersum* across the Liangzi Lake; 2) elucidated the correlations between environmental factors and genetic/epigenetic variation in *C. demersum*; and 3) identified suitable areas for species restoration.

## 2 Materials and methods

### 2.1 Sampling and DNA extraction

**Fig. 1:**
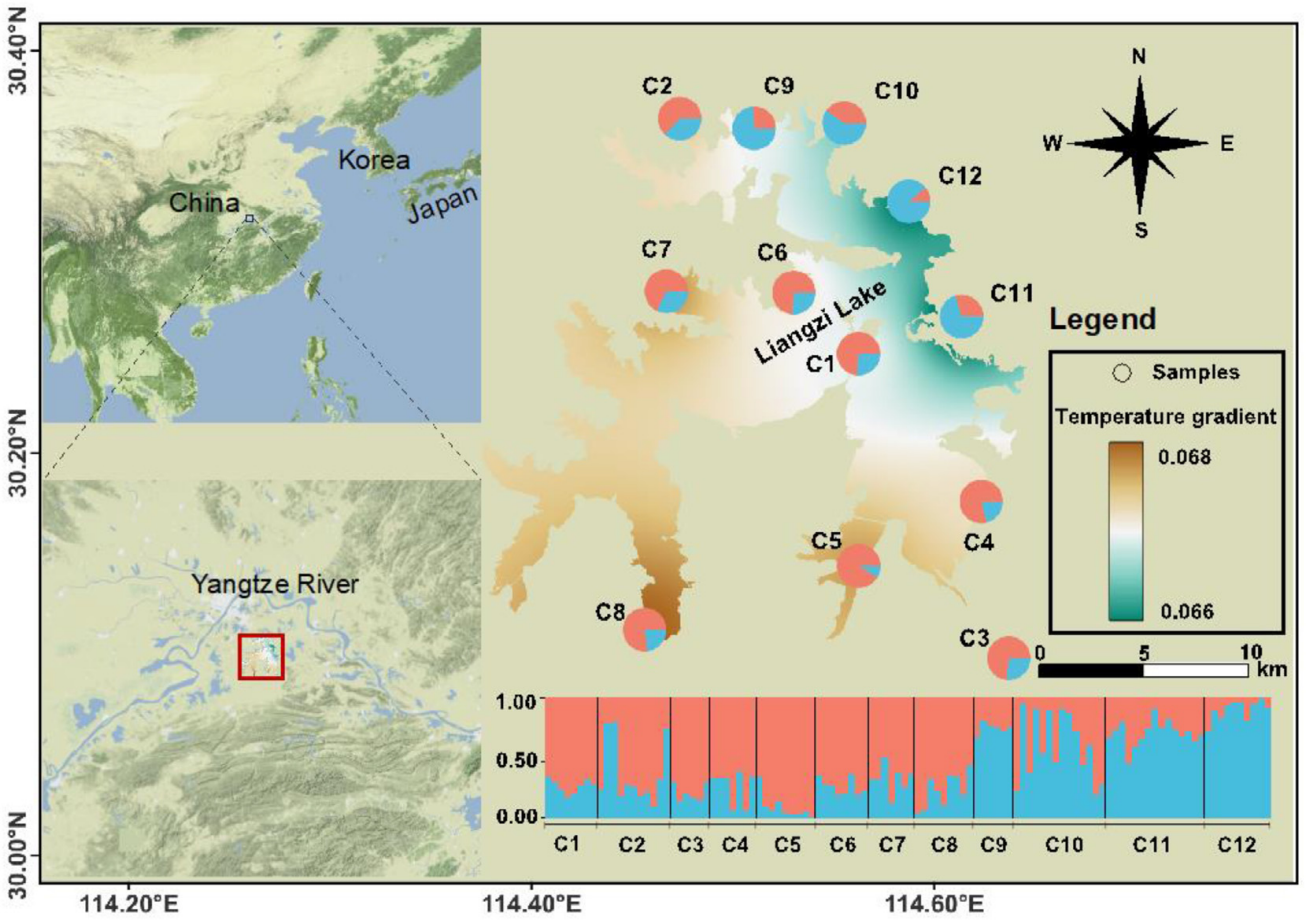
Sampling locations of 12 *Ceratophyllum demersum* populations across Liangzi Lake. The assignment of two major AFLP genetic clusters among *C. demersum* populations is shown in pie charts, with centroid transformed temperature scale of mean temperature of coldest quarter (MTCoQ) as a based map.

Samples of *Ceratophyllum* *demersum* were exhaustively searched for across the study area in July and December 2019. A total of 110 individuals were obtained from 12 sites (Fig. 1 and Table 1). The sampled populations were separated at a Euclidean distance of ∼5 km, and individuals were separated from each other by 20 m. Young leaves at the same development stage were collected, immediately dried in silica gel, and stored at −20 °C for further use. Total genomic DNA was extracted from approximately 20 mg of dried tissue using a Plant Genomic DNA Rapid Extraction kit (Tsingke, Beijing, China). The quality and quantity of genomic DNA was checked on a 1% TAE-agarose gel and measured by UV spectrophotometer (NanoDrop, Thermo Fisher Scientific, Waltham, MA, USA).

**Table 1:** Details of the sample sites, genetic diversity, and epigenetic variation for 12 populations of *Ceratophyllumdemersum* in Liangzi Lake.

					Genetic diversity			Epigenetic diversity		
	Code	Latitude	Longitude	N	*H*_S_	*I*	*PPL* (%)	e*H*_S_	e*I*	e*PPL* (%)
	C1	30.249	114.562	8	0.185	0.243	44.93	0.224	0.341	66.02
	C2	30.366	114.474	11	0.256	0.355	71.01	0.252	0.396	85.30
	C3	30.176	114.623	6	0.208	0.261	48.65	0.216	0.325	59.52
	C4	30.098	114.637	7	0.222	0.292	57.35	0.214	0.329	65.06
	C5	30.144	114.563	9	0.138	0.195	43.48	0.212	0.326	65.78
	C6	30.280	114.530	8	0.237	0.318	62.53	0.247	0.371	69.64
	C7	30.280	114.467	7	0.234	0.306	59.21	0.157	0.241	46.99
	C8	30.112	114.456	9	0.223	0.304	61.28	0.151	0.240	52.53
	C9	30.361	114.511	6	0.333	0.410	72.05	0.263	0.390	69.64
	C10	30.364	114.555	14	0.340	0.476	91.93	0.275	0.426	89.16
	C11	30.268	114.614	15	0.345	0.481	90.89	0.308	0.468	92.29
	C12	30.325	114.587	10	0.374	0.497	88.41	0.335	0.495	88.67
**Mean**					0.258	0.345	65.98	0.237	0.362	70.88

Abbreviations: N, sample numbers; *H*_S_, Nei's gene diversity; e*H*_S_, Nei's gene diversity of epigenetic variation; *I*, Shannon's information index of genetic diversity; e*I*, Shannon's information index of epigenetic variation; *PPL* (%), percentage of polymorphic loci; e*PPL* (%), percentage of epigenetic polymorphic loci.

### 2.2 AFLP and MSAP genotyping

AFLP and MSAP molecular markers were used to evaluate the genetic and epigenetic diversity of *Ceratophyllum* *demersum*. The detailed procedures for AFLP and MSAP analysis and statistical analysis are described in Supporting information.

An initial selective PCR of eight individuals across four populations was carried out with 12 primer combinations; only primers that provided clear, reproducible bands with sufficient polymorphic variation between populations were used in the analysis. The six most informative primer combinations (E-ACA/M-CAA, E-ACT/M-CAC, E-ACT/M-CTT, E-AGC/M-CAA, E-AGC/M-CTC, and E-AGC/M-CTT) were retained for AFLP analysis, and six primer pairs (E-ACA/M-TTA, E-ACA/M-TTG, E-ACT/M-TTA, E-ACT/M-TTG, E-AGC/M-TTA, and E-AGC/M-TTG) were retained for MSAP analysis. To estimate the error rate in AFLP and MSAP genotyping, we randomly choose 12 DNA samples to duplicate the AFLP and MSAP procedures.

### 2.3 Environmental variables

During the assessment of habitat heterogeneity, 43 environmental variables were analyzed (Table S1). The stratum of the environmental gradient was examined based on the Euclidean distances of environmental variables at each sampling site. Water quality were determined in-situ with a multiparameter probe (YSI Company, Yellow Springs, OH, USA) and the pollutants were measured using the method recommended by the National Groundwater Analysis Standard Procedure (GB 3838-2002). The concentrations of six heavy metal elements (As, Cd, Pb, Se, Zn, and Cu) in the water samples were analyzed by inductively coupled plasma-mass spectrometry (ICP-MS, NexION2000, PerkinElmer, OH, USA). Distance to the nearest village (DTV), distance to the road (DTR), and number of populations in the village (NHV) were acquired using QGIS v.3.22 (http://www.qgis.org) and through field surveys by locals. Moreover, the global climate and weather database from Worldclim v.2.1 (http://worldclim.org) was used to extract 19 bio-climates attributes at a 30-arc-second resolution. Previous studies indicated that multicollinearity in variables might lead to the overfitting of models. To reduce the multicollinearity, the temperature (Bio1–11) and precipitation (Bio12–19) values were divided into two groups and the Pearson correlation coefficients were calculated. One variable in each pair with a Pearson's correlation coefficients (*r*) > 0.8 was eliminated. As topographic slope strongly influences the local temperature, thereby influencing the thermal conditions of the habitat, the slope aspect of each sampling site was analyzed based on the attributes of the topography of Liangzi Lake from the Advanced Spaceborne Thermal Emission and Reflection Radiometer Global Digital Elevation Model v.3 (ASTER GDAM v.3).

### 2.4 Data analysis

The AFLP and MSAP genotypes were obtained from ABI Sequence Prism 377 using GENSCAN v.3.7 (Thermo Fisher Scientific, Waltham, MA, USA). Fragments between 100 and 500 bp were detected using GeneMarker v.2.2.2 software (Hulce et al., 2011). The polymorphic loci were confirmed manually and transformed into a binary 0/1 matrix as presence ‘1’ or absence ‘0’ of the bands. For MSAP datasets, the R package msap (Pérez-Figueroa, 2013) was used to calculate the types of genome methylations present. Loci were classified as either methylated-susceptible loci (MSL) or non-methylated loci, and a 0/1 binary matrix was also constructed where ‘0’ represents a non-methylated locus and ‘1’ represents a methylated locus. Indices of genetic diversity and epigenetic variation within populations were obtained from POPGENE v.1.3.2 (Yeh, 1999) assuming Hardy–Weinberg equilibrium, including the (i) Nei's gene diversity (*H*_S_ and e*H*_S_), (ii) mean Shannon's information index (*I* and e*I*), and (iii) percentage of polymorphic loci (*PPL* and e*PPL*).

Based on the Jaccard genetic similarity coefficient, a UPGMA dendrogram was constructed to divide the genetic groups among populations with the R package ape (Paradis and Schliep, 2019). The proportion of genetic variation among groups of populations (ø_CT_), among populations within groups (ø_SC_), and within populations (ø_ST_) was examined by the analysis of molecular variance (AMOVA) using the R package poppr (Kamvar et al., 2014). GenAlEx v.6.503 (Smouse and Peakall, 2012)was also used to characterize the genetic and epigenetic differentiation at each locus. The levels of statistical significance were determined after 9999 permutations.

The potential population structure of *Ceratophyllum* *demersum* was inferred from the AFLP dataset using the Bayesian clustering approach with STRUCTURE v.2.3.4 software (Pritchard et al., 2000). The analysis was performed using the admixture ancestry model with correlated allele frequencies; the number of clusters (*K*) was set to vary from 1 to 12, with each run having 5 × 10^6^ Markov chain Monte Carlo (MCMC) iterations following a burn-in period of 10^6^ steps. To identify the appropriate number of clusters of individuals, the likelihood values were calculated to partition the populations into groups based on different *K* values with STRUCTURE HARVESTER v.0.6.94 (Earl and VonHoldt, 2012).

To further investigate the potential relationships between genetic diversity, epigenetic variation, geographic distance, and environmental distance, a pair-wise distance matrix was constructed based on genetic and epigenetic indices, and the Bray–Curtis distance was calculated among samples. The Euclidean geographic distance and Euclidean environmental distance were also computed using the coordinates of sampling sites and environmental variables, respectively. A partial mantel test was performed using the R package vegan (Oksanen et al., 2007) with 9999 permutations for a significance test to detect the relationships between genetic, epigenetic, geographical, and environmental distances. Procrustes analysis was also performed with the same package to further reveal the correlations between environmental factors and genetic variation.

The importance of environmental variables that correlated with genetic diversity and epigenetic variation was accessed by Boruta feature selection “random forest” analysis (*P* < 0.05) using R package Boruta (Kursa et al., 2020). Random forest (RF) is a robust supervised learning algorithm that can be used for various tasks including evaluating the importance of variance and classification problems. The RF model is commonly used to predict the accuracy of classification when the aim variances are factors, but it can also be used to measure the relative importance of each feature in the prediction. Environmental variables ranked by the RF model in order of importance to genetic and epigenetic variation were determined over 1000 iterations and collected for downstream analysis.

To quantify the pure and combined effects of environmental variables on genetic and epigenetic variation, distance-based redundancy analysis (db-RDA) was performed using the vegan package. This multivariate ordination technique is used to test whether the variation of one independent variables explains the variance of another independent variable. Furthermore, the contributions of environmental variables were determined by variance partitioning analysis (VPA) and hierarchical partitioning (HP) using the multiple regression algorithm in the R package rdacca.hp (Lai et al., 2022).

To detect the genetic-environmental associations, we used latent factor mixed models (LFMM2) in the R package lfmm to detect the correlations between environmental variables and genotypic variation (Caye et al., 2019). This method is efficient for dealing with false discovery rates, sampling design limitations, and spatially autocorrelated populations. The original genetic and epigenetic genotype data were used, and environmental values associated with genetic and epigenetic variation were scaled and centered for this analysis. We set *K* = 2 then ran 50,000 iterations, with 50% burn-in, and 10 repetitions. Significant genetic and epigenetic loci were retained with thresholds of *P* < 0.05.

To identify putative environmental factors that were correlated with genetic clusters, we analyzed whether the environmental variables could be used as predictors to divide the genetic clusters of –*Ceratophyllum* *demersum* populations. An RF model with 10-fold cross-validation was constructed with the ‘rfcv’ function in the R package randomForest (Liaw and Wiener, 2002). The minimum cross-validation error was obtained when using the correlated environmental variables detected above. Therefore, we choose this value as a potential predictor in clustering the genetic groups of all populations.

Other than the classification methods used above, we further investigated the feasibility of predicting genetic clusters from environmental variables using multi-layer perceptron artificial neural networks (MLP-ANNs) in R package neuralnet (Günther and Fritsch, 2010). The approach consists in training an MLP-ANN to predict genetic clusters based on a set of environmental variables, such as water and ambient temperatures.

Furthermore, as the pattern of genetic clusters may show a non-linear correlation with environmental factors, generalized additive models (GAMs) were employed to fit the environmental variables to the population clusters detected above using the ‘ordisurf’ function in vegan. GAM models allow for a linear or nonlinear fit of environmental variables to the detected genetic structure. Without a transformed environmental distance to fit the analysis, these models fit the environmental data as a smooth response over the genetic structure accounting for both axes.

Next, to determine the degrees of environmental contributions at the genetic diversity and epigenetic variation levels, the partial least squares path model (PLS-PM), which is widely used to study complex multivariate relationships among variables, was used to quantify the direct and indirect effects of environmental variables on genetic and epigenetic variation. The PLS-PM does not require any distributional assumptions about the data, which are usually difficult to meet in natural ecosystem. We established a model based on the expected relationships and key drivers among environmental variables, genetic variation, and epigenetic variation with the R package plspm (Ravand and Baghaei, 2016). In the model, we compiled variables that were highly linear with respect to bio-climates as latent variables and performed nonparametric bootstrapping validation (1000 resamples) to estimate the precision of the parameters. A bootstrap confidence interval of 95% was used to judge whether estimated path coefficients were significant. The final model was chosen among all constructed models based on the goodness of fit (GoF; > 0.7).

Suitable areas for species restoration were chosen based on the genetic diversity and epigenetic variation level using R package prioritizr (Hanson et al., 2017). This package uses mixed-integer linear programming (MILP) techniques to provide a flexible interface for building and solving restoration planning problems. Prioritizr supports a broad range of constraints that can be used to address restoration planning problems based on the specific needs of a restoration planning exercise. In addition, prioritizr finds solutions in a much shorter period of time than other programs. We developed trade-offs restoration prioritizations to identify priority areas for protected area establishment based on the degrees of which environmental variables contribute to genetic diversity and epigenetic variation. Our aim was to ensure that 20% of total genetic diversity and epigenetic variation among the surveyed populations are conserved (Failler et al., 2019). Environmental variables that correlated to genetic and epigenetic indices were used as penalty and constraint factors to solve the restoration planning problems.

## 3 Results

### 3.1 Environmental gradient over the sampling sites

**Fig. 2:**
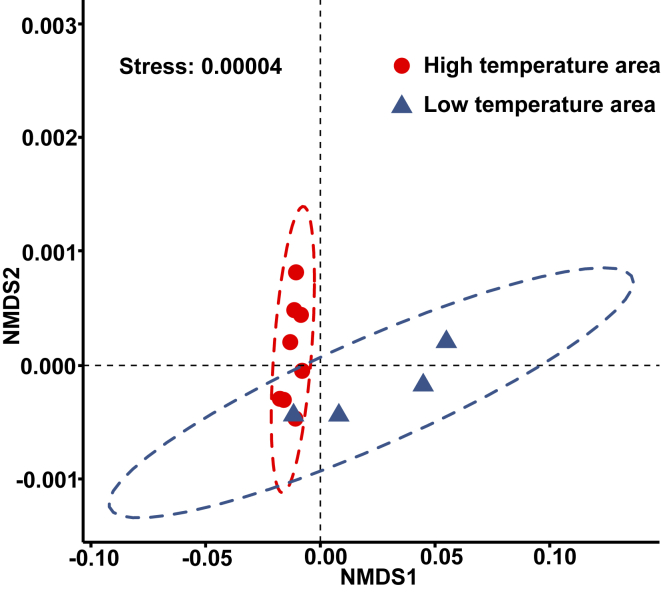
The results of NMDS allows two clear environmental groups based on the Euclidean distance at each population sites of *Ceratophyllumdemersum*.

We captured a substantial environmental gradient over the sampling area (Stress = 0.00004, Fig. 2) according to the results of NMDS analysis. In addition, WT, AMT, MTWeQ, and MTCoQ showed an obvious gradient at a relatively fine geographic waterbody scale.

### 3.2 Genetic diversity and epigenetic variation in the populations

Using six AFLP primers, a total of 483 bands were obtained from 12 populations of *Ceratophyllum* *demersum*. The number of bands generated by different primer combinations ranged from 70 to 89 (Table S2). At the species level, the *PPL*, *H*_S_ and *I* were 65.98%, 0.258 and 0.345, respectively. At the population level, the *PPL* of each population ranged from 43.48% (C5) to 91.93% (C10), with an average of 63.94%. Population C12 (*H*_S_ = 0.374, *I* = 0.476, *PPL* = 88.41%) exhibited the highest level of genetic diversity, and population C5 (*H*_S_ = 0.138, *I* = 0.195, *PPL* = 43.48%) showed the lowest level of genetic diversity (Tables 1 and S4).

The number of bands generated by MSAP primer combinations ranged from 76 to 88 (Table S3). At the species level, the e*PPL*, e*H*_S_ and e*I* were 70.88%, 0.238 and 0.362, respectively, which was relatively higher than genetic diversity. At population level, population C12 showing the highest level of genomic methylation (e*H*_S_ = 0.335, e*I* = 0.495, e*PPL* = 88.67%) and population C8 (e*H*_S_ = 0.151, e*I* = 0.240, e*PPL* = 52.53%) showing the lowest (Tables 1 and S4).

**Fig. 3:**
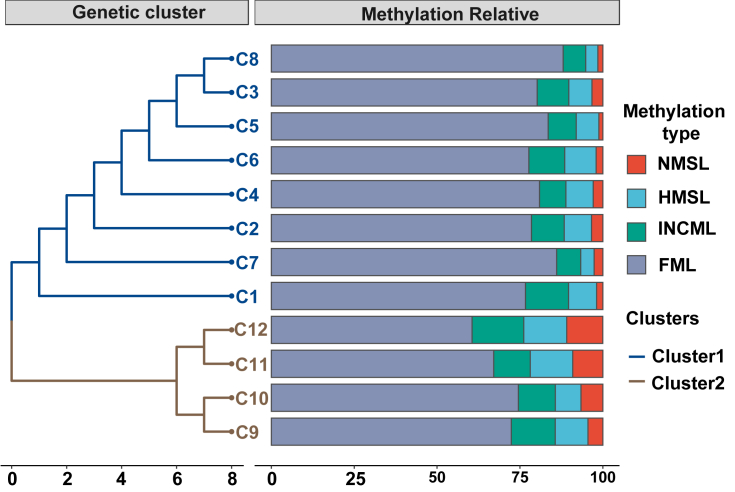
Dendrograms generated using unweight pair group method with arithmetic average (UPGMA), showing genetic relationships between 12 *Ceratophyllumdemersum* populations based on the Jaccard genetic similarity coefficient. Relative abundance of population-based methylation among *C. demersum* samples are shown in bar plot. NMSL represented non methylation, HMSL depicted the hemi-methylated proportion, INCML indicated the inner cytosine methylation and FML was classified as full methylated state of DNA.

The population-based UPGMA tree contains two genetic clades among the *C. demersum* populations based on the Jaccard genetic similarity coefficient (Fig. 3). In addition, the methylation types of the loci varied among the genetic clades: populations C9, C10, C11, and C12 exhibited lower levels of full methylation types (mean FML = 68.61%) and higher levels of unmethylated types (mean NMSL = 7.78%) (Fig. 3). Conversely, populations C1–C8 showed lower FML (mean FML = 81.42%) and NMSL (mean NMSL = 2.37%) (Table S5). Non-hierarchical analysis of molecular variance (AMOVA) of the genetic data revealed genetic differentiation within populations of 89%, indicating strong genetic variation among populations. Hierarchical AMOVA attributed 9.1% of total variance to differences between two groups and 6.2% to differences between groups among populations, whereas most variance was partitioned within populations (84.6%) (Table S6).

### 3.3 Population genetic structure

In the population structure analysis, the highest likelihood at *K* = 2 was revealed based on Bayesian assignment (Figs. 1 and S1), and PCA results clarified the genetic groups in *Ceratophyllum* *demersum* (Fig. S2). Therefore, the clades within the *C. demersum* populations consisted of two clusters: populations from low-temperature areas (C9–C12) comprised cluster 1, whereas populations from high-temperature areas (C1–C8) comprised cluster 2.

### 3.4 Correlations among genetic diversity, epigenetic variation, and environmental variables

**Fig. 4:**
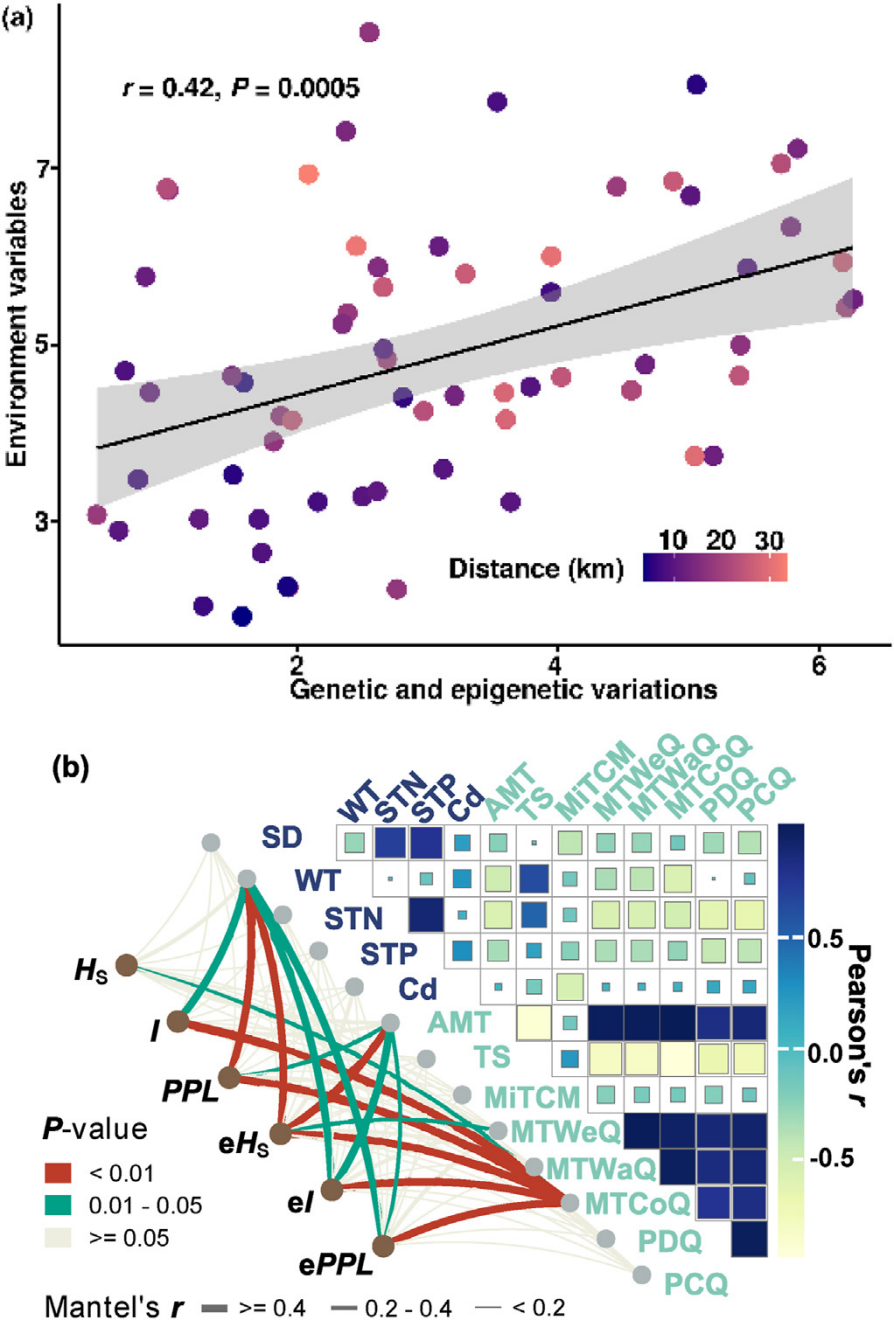
Correlations among genetic distance, geographic distance, and Euclidean environmental distance of *Ceratophyllumdemersum* populations. (a) Pearson correlations between genetic/epigenetic variables and environment variables, geographic distance among populations are shown in colored gradient plot. (b) Partial mantel test detected the coefficient of genetic diversity and epigenetic variation with environmental variables.

A partial mantel test revealed a significant pattern of correlation of genetic and epigenetic variation with environmental variables (*r* = 0.42, *P* < 0.01) (Fig. 4a). We filtered 13 environmental variables that accounted for over 1% of the estimated importance of genetic and epigenetic indices using the RF model (Fig. S3a and S3b). Environmental variables including WT, AMT, MTWeQ, and MTCoQ were strongly correlated with the genetic diversity and epigenetic variation indices (Mantel's *r* > 0.2, *P* < 0.05) (Fig. 4b). Procrustes analysis of the NMDS results described above further confirmed the strong associations between genetic clusters and the environmental stratum (*M*^2^ = 0.514, *P* = 0.005) (Fig. S4).

Of the core variants detected by LFMM, 6 genetic loci were associated with environmental variables (FDR < 0.01) (Fig. S5), indicating that adaptation to temperature has primarily evolved as a result of selection acting on regulatory environment changes. In particular, 9 most significant epigenetic outlier was detected by LFMM (FDR < 0.01), where locus77, locus 210, locus235 and locus405 were strongly associated with AMT, locus63, locus235, and locus405 were associated with MTWeQ, locus61 and locus 182 were associated with MTCoQ (Fig. S6).

**Fig. 5:**
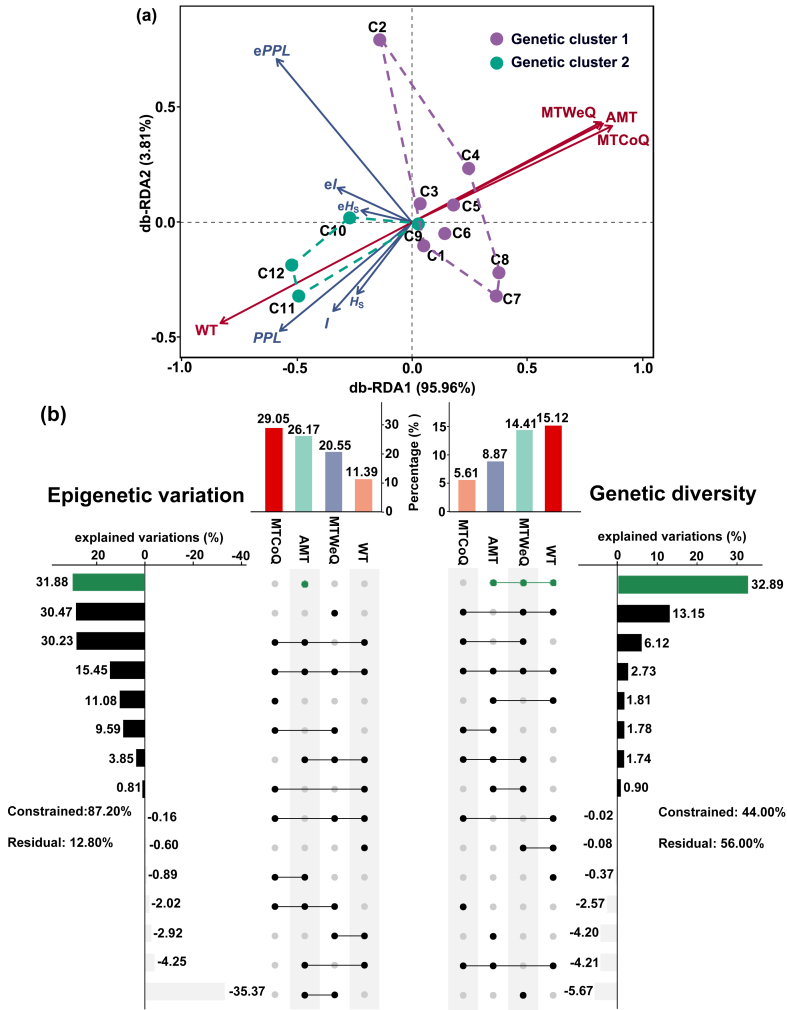
Distance-based redundancy analysis plot demonstrated the ordination of genetic and epigenetic indices responses from *Ceratophyllumdemersum* under the influences of four environmental variables (a) and histogram of the relative contributions (b) of environmental variables in contrast to genetic diversity and epigenetic variation in Liangzi Lake. The dot matrix and the corresponding bar show the values of pure and shared contributions. Negative values due to adjustment of R^2^ mean negligible but included in the computation of the total contribution of four environmental variables. Environmental variables with largest individual and overlapping contributions are highlighted in forest green. The residuals value represents the percentage of unexplained by the environmental variables.

In the distance-based redundancy analysis (db-RDA) results, the first two axes explained 99.77% of the variances, accounting for 95.96% (axis 1) and 3.81% (axis 2), respectively, of the genetic and epigenetic variation (Fig. 5a). WT had a vital effect (individual effect = 15.12%) and made the largest contribution to genetic diversity. In fact, WT, MTWeQ, and AMT shared the effects on genetic diversity, as revealed by variance partitioning analysis (explained variations = 32.89%). However, although MTCoQ showed the highest positive individual effect on epigenetic variation, as revealed by hierarchical partitioning (individual effect = 29.05%), AMT overshadowed this influence (explained variation = 31.88%) and explained the largest portion of measured epigenetic variation (Fig. 5b).

**Fig. 6:**
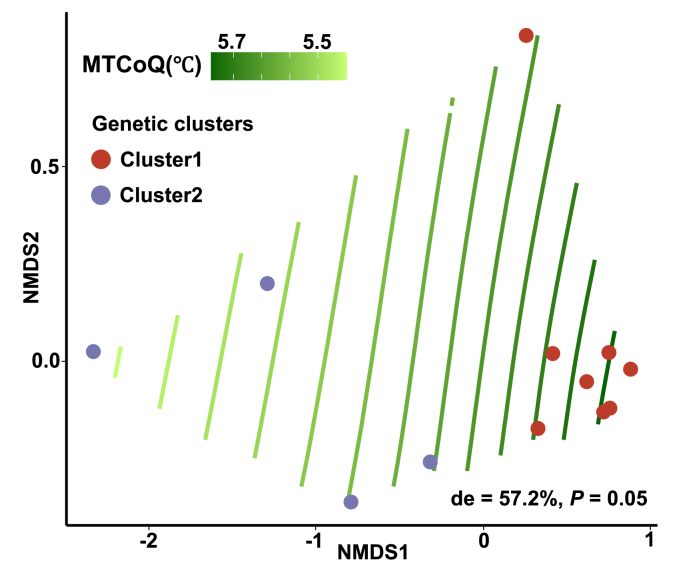
Results from the generalized additive models fitting measured MTCoQ across the nonmetric multidimensional scaling (NMDS) ordination of detected population structure. Points are colored by *Ceratophyllum demersum* populations. Green splines show the fit of the MTCoQ from high values (dark green) to low values (light green) over the ordination. The gradient splines would be parallel if the relationship between MTCoQ and genetic cluster is linear. Nonlinear relationships between MTCoQ and population structure are represented by curved splines. ‘de’ shows the deviance which explained by the respective model.

The genetic clades retained among populations essentially matched the environmental gradient measured in our NMDS analysis: that is, we detected a non-negligible association between genetic clades and environmental groups. Furthermore, MTCoQ was identified as an environmental predictor to distinguish the two genetic clusters, as it showed the lowest cross-validation error in our RF model (Fig. S3c). The MLP-ANN-based approach for predicting the genetic clusters also identified MTCoQ was the key factor to divide the clusters retained in *C. demersum* populations (Fig. S7). Besides, GAMs model further confirmed that MTCoQ was a strongly determinant of the genetic clusters among populations, which explained over half of deviation within our model (Fig. 6).

**Fig. 7:**
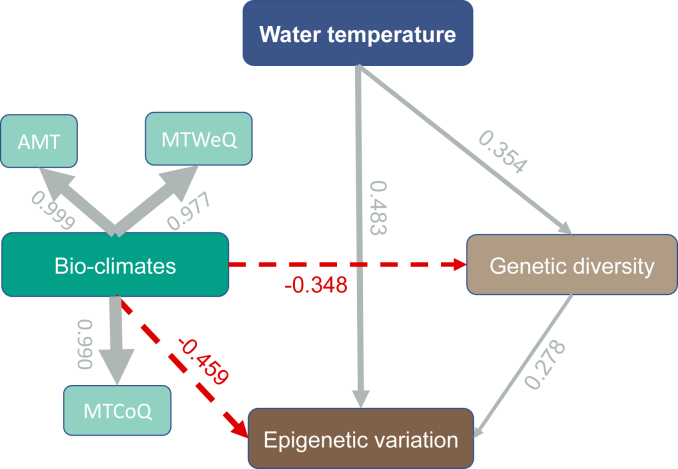
Effects of water temperature and bio-climates on the genetic diversity and epigenetic variation of *Ceratophyllum demersum*. Bio-climates is a latent variable compiled with AMT, MTWeQ and MTCoQ. The line width is proportional to the effect strength. Numbers adjacent to arrows are standardized path coefficients. Continuous and dashed arrows indicate positive and negative relationships, respectively.

Our PLS-PM model added to the robustness of our data, as suggested by the goodness of fit (GoF = 0.82). PLS-PM analysis determined that WT had a positive influence on variation, accounting for 65.8% (*R*^2^ = 0.658) of variation in genetic diversity (path coefficient = 0.354, *P* < 0.05) and epigenetic variation (path coefficient = 0.483, *P* < 0.05) (Fig. 7). By contrast, bio-climates negatively influenced the genetic diversity (path coefficient = −0.348) and epigenetic variation (path coefficient = −0.459), contributing 71.4% of the variations in these parameters (*R*^2^ = 0.714). Finally, genetic diversity was found to positively drive epigenetic variation (path coefficient = 0.278, *P* < 0.05) according to our model.

### 3.5 Species restoration areas

**Fig. 8:**
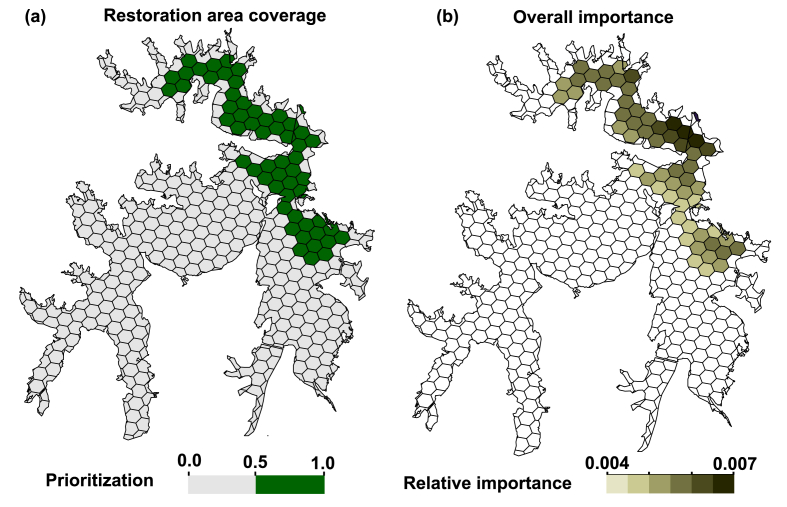
A trade-offs restoration scenario includes 20% of genetic diversity and epigenetic variation of *Ceratophyllumdemersum* populations are generated. (a) The restoration units are selected when MTCoQ are used as constraints, genetic diversity and epigenetic variation are placed as decision variables. (b) Relative importance of restoration units are shown in color ramp.

The lake area was divided into priority and relatively important areas for species restoration based on genetic diversity and epigenetic variation. 57 units were selected as restoration areas, and population C1 and C9–C12 are located at prioritized restoration areas (Fig. 8a), which comprised the genetic structure of *Ceratophyllum* *demersum*. Ferrier analysis further quantified the relative importance units, and results showed that C12 is located in the most important restoration area (Fig. 8b).

## 4 Discussion

### 4.1 The geographic pattern of genetic and epigenetic variation

Unrooted aquatic plants usually possess the capacity for a high level of dispersal but can show distinct genetic variation among populations. The pattern of genetic variation and epigenetic variation is often driven by geographic and environmental influences exerted by geographic events, anthropogenic activities, and climatic oscillations (Orsini et al., 2013; Wang and Bradburd, 2014). Geographic isolation is usually considered to be required for allopatric speciation, as geographically isolated populations usually represent unique habitat specialists due to their gradual adaption to local conditions (Coyne, 2007; Karbstein et al., 2021), therefore, genetic variation reflects not only differentiation but also ecological divergence among populations.

At the fine geographic scale, especially in lakes, species dispersal usually overrides the restriction of geographic barriers, particularly for unrooted aquatic plants. In the current study, genetic and epigenetic variation were not correlated with geographic distance but were positively correlated with environmental distance. These results at least partially confirm the isolation by environment hypothesis and are in agreement with the results of empirical genetic studies on terrestrial plants (Herrera et al., 2017).

### 4.2 Correlations among genetic diversity, epigenetic variation, and environment variables

The fine-scale water environment is hierarchical and highly vulnerable to changes in climate, as the ever-changing environment plays a pivotal role in driving the genetic diversity and epigenetic variation of aquatic plant species (Santamaria, 2002; Schulz et al., 2014). Due to the potential inheritance and rapid adjustment of adaption, it is essential to evaluate the genetic diversity of a species as well as epigenetic variation level even at fine-scale levels of topographic and microclimatic variation (Foust et al., 2016; Zheng et al., 2019). Most previous genetic studies on aquatic plants have neglected to characterize the correlations among environmental variables, species genetic diversity, and epigenetic variation. Therefore, evidence for the association of abiotic factors with genetic and epigenetic variation remains scarce (Hughes and Stachowicz, 2009). Indeed, our study discovered a clear environmental gradient in Liangzi Lake that is shaped by temperature factors. As expected, genetic and epigenetic variation among *Ceratophyllum* *demersum* populations showed a correlative relationship, which in line with this environmental gradient. The air temperature scale has created a scenario in which higher-temperature areas maintain lower genetic and epigenetic variation, and lower-temperature areas maintain higher levels of genetic and epigenetic variation. Our genetic and epigenetic analysis of *Vallisneria natans* in the same area (unpublished data) further validates these results, by which higher water temperature areas also maintains lower genetic and epigenetic variation. Besides, by using the published data, we also detected negative correlations between AMT and the genetic diversity using the published data in *Hydrocotyle vulgaris* (Wang et al., 2020).

For clonal macrophytes, although studies have revealed a concave-up and significant relationship between intra-population genetic diversity and epigenetic variation (Wang et al., 2020), the contribution of genetic diversity to epigenetic variation has still not been described clearly, as epigenetic variation is, at least in part, a subsidiary effect of genetic variation (Richards et al., 2012). Our PLS-PM analysis revealed the direct effects of genetic diversity on epigenetic variation. However, while comparing the effects driven by environmental variables, we determined that such an influence can be overwhelmed during the slow adaption of plants to variations in environmental conditions.

### 4.3 The role of temperature in genetic variation and epigenetic variation

A major challenge in population genetic analysis is the difficulty of distinguishing genetic divergence caused by historical events from those due to anthropogenic disturbances (Inostroza et al., 2016; McKown et al., 2014). Although the Liangzi Lake maintains the highest diversity of aquatic plants in Yangtze River basin due to its complex topography, the environmental conditions in this area have been rapidly changing in recent years (Zhang et al., 2019). Seasonal floods, especially in 2016 and 2020, have diminished the number of aquatic plant species. Our field investigation of aquatic plant diversity in this area from 2019 to 2021 demonstrated that the populations of aquatic plants could quickly recovered from floods. In addition, the population of *Ceratophyllum* *demersum* decreased sharply in 2021 compared to 2019, right at the start of a 10-years fishing ban in key areas of the Yangtze River basin. As *C. demersum* is eaten by herbivorous fish species and crabs, perhaps the reduced *C. demersum* population was due to the disequilibrium between aquatic herbs and herbivorous fish in this environmental system.

Temperature-imposed selection usually leads to the local adaption of species across a large geographic scale, where temperature gradients often result in spatial divergence, thereby leading to the population divergence (Medina et al., 2021). Moreover, the different levels of genetic diversity among populations throughout the thermal regime can be beneficial to the consumers, which further influences their genetic diversity and helps shape the population structure in return (Farleigh et al., 2021; Ruff et al., 2011). Therefore, how intraspecific genetic variation is being shaped in the context of temperature range where such an environmental gradient exist is still an open question.

In our analysis, patterns of genetic variation across a range of the species illustrate how temperature affects genetic and epigenetic variation. Genetic and epigenetic loci were also found to associated with temperature variables. More importantly, we found that in our study area, located in a subtropical region of China, water temperature had positive effects on genetic and epigenetic variation, while air temperature had negative effects on these traits. Populations located in areas with lower ambient temperatures exhibited greater genetic and epigenetic variation, whereas populations located in relatively higher-temperature areas showed lower genetic and epigenetic variation. This distribution of genetic and epigenetic variation in lakes challenges the notions that high-temperature areas maintain greater biodiversity than colder areas. However, our results are consistent with the findings of a study on the effects of evolutionary capacity on total genetic variation in two reef-building corals in response to natural selection: warm temperatures drove corals lacking evolutionary potential to rapid functional extinction due to the low genetic variation within their populations (Walsworth et al., 2019).

In lake areas, the expansion of macrophytes usually leads to an increase in dissolved oxygen levels. High oxygen levels maintain the water temperature at a lower level than in sites where the dissolved oxygen content is low. In other words, higher-biodiversity areas in aquatic systems can maintain the water temperature at lower levels (Hamberg et al., 2020; Jane et al., 2021). In addition, air and water temperatures are not related in all lakes in all climate regions; the site temperature usually depends on the depth of the lake, especially when it is extremely shallow. In our study area, the water temperature is maintained well above 0 °C. However, the air temperature in the water can be lower than 0 °C, and therefore, the linear and non-linear relationships between air and water temperature would disappear in this area. Since the global average surface air temperature is expected to increase by 3 °C by the end of this century (Friedlingstein et al., 2010), surface water temperatures in lakes will be likely increased as the air temperature increases. Although the changes in water temperature may be smaller than the changes in air temperature, even these slight changes in water temperature might affect the genetic and epigenetic variation of aquatic plants to some extent.

Moreover, as the genetic structure detected among *Ceratophyllum* *demersum* populations was separated based on geography but on temperature, our results emphasize the importance of temperature in shaping the pattern of genetic variation of aquatic plants at a fine geographic scale. Meanwhile, empirical studies have mainly focused on large geographic scales and mitigated the temperature influences as detailing the habitats variables can be a daunting task (Liang et al., 2022). Our analysis largely eliminated the effects of geography on genetic analysis and filled a knowledge gap about the effects of temperature on the genetics of aquatic plants, increasing our understanding of the robust relationships among genetics, epigenetic variation, and environmental variables.

### 4.4 Implications for restoration

The genetic diversity of a species, which reflects its capacity of species for short-term ecological adaptation and long-erm evolutionary changes, is generally taken into consideration in designing restoration sites (Teixeira and Huber, 2021). In regard to aquatic species restoration, however, little is known about genetic variation and species-sites interactions, especially in regard to restoration projects aimed at mitigating the effects of extreme climate events or anthropogenic influences (Forester et al., 2022). Furthermore, genetic diversity is considered to be crucial for adaptation of species to unforeseen changes in climate and for maintaining resilience to abiotic and biotic stress. The Liangzi Lake is a newly protected area and has thus been buffered from anthropogenic threats other than climate change since 2020. As protected areas should conserve not only intraspecific genetic diversity but also habitat heterogeneity, identifying potential relationships between genetic and epigenetic variation and environmental variables, and linking these relationships to fitness, would offers the opportunity to construct restoration portfolios even when environmental pressures are difficult to measure (Bay et al., 2017). Based on our analysis, the suitability of areas for species restorations depends not only on the levels of genetic diversity and epigenetic variation but also on incremental temperature benefits. Trade-offs must be made among genetic diversity, epigenetic variation, and temperature when designing prioritized restoration areas.

## 5 Conclusion

Our genetic analysis of *Ceratophyllum* *demersum* in the Liangzi Lake delineated cryptic correlations among genetic diversity, epigenetic variation, and environmental variables and set the bias for estimate temperature in shaping the geographical pattern of genetic variation of clonal aquatic herbs in thermal gradients. Environmental variables drive species adaption in conjunction with genetic diversity and epigenetic variation. Genetic and epigenetic variations among populations can override the influences of geographic isolation when significant environmental gradients exist. Moreover, a species restoration strategy should take the temperature into account even at a fine scale, especially for the aquatic plants, as their suitable habitats are usually confined to a restricted water environment.

## Author contributions

H-W.H. and Y-X.L. conceived and designed the research; Y-X.L. designed and conducted the experiments, collected the data, performed the analyses, generated the figures and write the manuscript. M-L.X., X-Y.Z. and X-Z.W. revised the manuscript and edited the language. All authors approved the final manuscript.

## Data accessibility

All codes and polymorphism raw data are openly available from the Github repository: https://github.com/yixian185/For-FEE.

## Declaration of competing interest

The authors declare that they have no known competing financial interests or personal relationships that could have appeared to influence the work reported in this paper.
