# Supplementary material for: Temperature is a cryptic factor shaping the geographical pattern of genetic variation in Ceratophyllum demersum across a subtropical freshwater lake
Source: Plant Divers. 2023 Aug 19;46(5):630–9. doi: 10.1016/j.pld.2023.08.002 (PMC11403116; doi:10.1016/j.pld.2023.08.002)
Supplement: Multimedia component 1 [file mmc1.docx]

**Supporting Information**

**Detailed Methods**

**AFLP and MSAP genotyping**

AFLP and MSAP genotyping were carried out according to the protocols recommended by Vos and modified by Baurens (Baurens et al., 2008; Vos et al., 1995). For the AFLP analysis, 100 ng DNA in a total volume of 40 μL was digested using a cocktail composed of 0.15 μL *EcoR*I (20 U·µL^-1^) and 0.3 μL *Mse*I (20 U·µL^-1^) (New England Biolabs, Ipswich, MA, USA) for 1.5 hr at 37 ℃. For MSAP analysis, the DNA template was digested using *EcoR*I and a pair of isoschizomeric enzymes in place of *Mse*I: *Hpa*II and *Msp*I. Both of these enzymes recognize the CCGG sequence but show different sensitivities to the methylation status of external or internal cytosine residues, thereby reflecting the types of methylation based on the the lengths of the digested bands.

The products were visualized by 1.5% agarose gel electrophoresis to ensure that the templates were completely digested. Subsequently, 10 µL of a mixture which containing 3 µL *EcoR*I adaptor (5 µM), 3 µL *Mse*I/*Hpa*II/*Msp*I adaptor (50 µM), 1 µL T4 ligase buffer (10×), 0.5 µL T4 DNA ligase (400 U·µL^-1^) (New England Biolabs, Ipswich, USA), and 2.5 µL dd H_2_O was added to the samples. The ligation reaction was incubated overnight at 4 ℃ in an ABI Veriti 96-well thermocycler (Thermo Fisher Scientific, Waltham, MA, USA), and the products were diluted 10-fold in dd H_2_O for the amplification reaction.

For pre-selective DNA amplification, 5 µL of the diluted DNA restriction-ligation product was added to a 20 µL cocktail containing 2 µL 10×Ex-Taq buffer, 1.2 µL Mg^2+^ (25 mM), 1.6 µL dNTPs (2.5 mM), 1 µL primer (10 µM·L^-1^) E+A (Invitrogen, CA, USA), 1 µL primer (10 µM·L^-1^) M+C/T (Invitrogen, CA, USA), and 0.1 µL Ex-Taq (5 U·µL^-1^). PCR was performed at 65 °C for 5 min, followed by 30 cycles of 30 s of denaturation at 94 °C, 30 s of annealing at 56 °C, and 1 min of elongation at 72 °C, and a final step of 5 min at 72 °C step for complete extension. For selective PCR amplification, 25 µL 10-fold diluted pre-PCR product was added to a 15 µL cocktail containing 2 µL 10× Ex-Taq buffer, 2 µL Mg^2+^ (25mM), 1.6 µL dNTPs (2.5 mM), 0.2 µL 5-FAM**EcoR*I (10 µM·L^-1^), 1.2 µL *Mse*I/*Hpa*II/*Msp*I (10 µM·L^-1^), and 0.1 µL Ex-Taq (5 U·µL^-1^). The PCR parameters were 2 min at 94 °C, 13 cycles of 30 s of denaturation at 94 °C, 30 s of annealing at 65 °C, and 1 min of elongation at 72 °C, but with the annealing temperature reduced every by 0.7 °C at each step; this was followed by an additional 23 cycles of 30 s of denaturation at 94°C, 30 s of annealing at 56°C, and 1 min of elongation at 72 °C, followed by a 5-min step at 72 °C. All reagents for PCR amplification were from Takara (Takara Biomedical, Beijing, China).

**Table S1. Environmental variables used in the assessment of habitat heterogeneity**

| Name | Abbreviation |
| --- | --- |
| Elevation | Elevation |
| pH | pH |
| Secchi depth | SD |
| Water-body temperature | WT |
| Conductivity | Cond |
| Total dissolved solids | TDS |
| Salinity | Sal |
| Dissolved oxygen | DO |
| Total nitrogen | TN |
| Total phosphorus | TP |
| Chemical oxygen demand | COD |
| Chlorophyll-a | *Chl*-a |
| Total nitrogen in sedements | STN |
| Total phosphous in sedements | STP |
| Arsenic | As |
| Cadmium | Cd |
| Lead | Pb |
| Copper | Cu |
| Zinc | Zn |
| Selenium | Se |
| Distance to Road | DTR |
| Distance to Village | DTV |
| Number of Human in Village | NHV |
| Slope | Slope |
| Bio1 (Annual mean temperature) | AMT |
| Bio2 (Mean diurnal range) | MDR |
| Bio3 (Isothermality) | Isothermality |
| Bio4 (Temperature seasonality) | TS |
| Bio5 (Maximum temperature of warmest month) | MaTWM |
| Bio6 (Minimum temperature of coldest month) | MiTCM |
| Bio7 (Temperature annual range) | TAR |
| Bio8 (Mean temperature of wettest quarter) | MTWeQ |
| Bio9 (Mean temperature of driest quarter) | MTDeQ |
| Bio10 (Mean temperature of warmest quarter) | MTWaQ |
| Bio11 (Mean temperature of coldest quarter) | MTCoQ |
| Bio12 (Annual precipitation) | AP |
| Bio13 (Precipitation of wettest month) | PWM |
| Bio14 (Precipitation of driest month) | PDM |
| Bio15 (Precipitation seasonality) | PS |
| Bio16 (Precipitation of wettest quarter) | PWeQ |
| Bio17 (Precipitation of driest quarter) | PDQ |
| Bio18 (Precipitation of warmest quarter) | PWaQ |
| Bio19 (Precipitation of coldest quarter) | PCQ |

**Table S2. Polymorphic bands obtained using six AFLP primer combinations**

| Primer combination | Sequence (5’- 3’) | Polymorphic band |
| --- | --- | --- |
| E-AGC/M-CTC | E: GACTGCGTACCAATTCAGC  M: GATGAGTCCTGAGTAACTC | 70 |
| E-AGC/M-CAA | E: GACTGCGTACCAATTCAGC  M: GATGAGTCCTGAGTAACAA | 79 |
| E-AGC/M-CTT | E: GACTGCGTACCAATTCAGC  M: GATGAGTCCTGAGTAACTT | 86 |
| E-ACT/M-CAC | E: GACTGCGTACCAATTCACT  M: GATGAGTCCTGAGTAACAC | 89 |
| E-ACT/M-CTT | E: GACTGCGTACCAATTCACT  M: GATGAGTCCTGAGTAACTT | 80 |
| E-ACA/M-CAA | E: GACTGCGTACCAATTCACA  M: GATGAGTCCTGAGTAACAA | 79 |

**Table S3. Polymorphic bands obtained using six MSAP primer combinations**

| Primer combination | Sequence (5’- 3’) | Polymorphic band |
| --- | --- | --- |
| E-ACT/M-TTA | E: GACTGCGTACCAATTCACT  M: GATGAGTCTAGAACGGTTA | 85 |
| E-ACT/M-TTG | E: GACTGCGTACCAATTCACT  M: GATGAGTCTAGAACGGTTG | 84 |
| E-AGC/M-TTA | E: GACTGCGTACCAATTCAGC  M: GATGAGTCTAGAACGGTTA | 76 |
| E-AGC/M-TTG | E: GACTGCGTACCAATTCAGC  M: GATGAGTCTAGAACGGTTG | 83 |
| E-ACA/M-TTA | E: GACTGCGTACCAATTCACA  M: GATGAGTCTAGAACGGTTA | 79 |
| E-ACA/M-TTG | E: GACTGCGTACCAATTCACA  M: GATGAGTCTAGAACGGTTG | 88 |

**Table S4. Genetic and epigenetic variation indices for 12 populations of *C. demersum***

|  | Code | Na | Ne | lambda | la | rbarD | eNa | eNe |
| --- | --- | --- | --- | --- | --- | --- | --- | --- |
|  | C1 | 1.449 | 1.275 | 0.875 | 9.525 | 0.044 | 1.660 | 1.370 |
|  | C2 | 1.710 | 1.382 | 0.909 | 16.399 | 0.048 | 1.853 | 1.394 |
|  | C3 | 1.487 | 1.286 | 0.833 | 3.832 | 0.016 | 1.595 | 1.360 |
|  | C4 | 1.574 | 1.305 | 0.857 | 3.278 | 0.012 | 1.651 | 1.341 |
|  | C5 | 1.435 | 1.186 | 0.889 | 9.055 | 0.044 | 1.658 | 1.342 |
|  | C6 | 1.625 | 1.337 | 0.875 | 2.174 | 0.007 | 1.696 | 1.413 |
|  | C7 | 1.592 | 1.328 | 0.857 | 2.878 | 0.010 | 1.467 | 1.252 |
|  | C8 | 1.613 | 1.322 | 0.889 | 6.536 | 0.022 | 1.525 | 1.229 |
|  | C9 | 1.721 | 1.478 | 0.833 | 3.564 | 0.010 | 1.696 | 1.448 |
|  | C10 | 1.919 | 1.527 | 0.929 | 10.227 | 0.023 | 1.892 | 1.436 |
|  | C11 | 1.909 | 1.549 | 0.933 | 2.774 | 0.006 | 1.923 | 1.511 |
|  | C12 | 1.884 | 1.581 | 0.900 | 2.442 | 0.006 | 1.887 | 1.581 |
| Mean |  | 1.660 | 1.380 | 0.882 | 6.057 | 0.021 | 1.709 | 1.390 |

Abbreviations: Na, observed number of alleles; eNa, epigenetic observed number of alleles; Ne, effective number of alleles; eNe, epigenetic effective number of alleles; lambda, Simpson’s index (Simpson, 1949); Ia, index of association for each population factor (Agapow and Burt, 2001); rbarD, standardized index of association for each population factor (Brown et al., 1980; Smith et al., 1993).

**Table S5.** **Rate of methylation types in 12 *C. demersum* populations**

| Species | NMSL | HMSL | INCML | FML |
| --- | --- | --- | --- | --- |
| C1 | 0.019 | 0.085 | 0.130 | 0.767 |
| C2 | 0.034 | 0.082 | 0.099 | 0.784 |
| C3 | 0.033 | 0.070 | 0.095 | 0.802 |
| C4 | 0.029 | 0.082 | 0.080 | 0.809 |
| C5 | 0.012 | 0.068 | 0.085 | 0.835 |
| C6 | 0.020 | 0.095 | 0.108 | 0.777 |
| C7 | 0.027 | 0.040 | 0.073 | 0.861 |
| C8 | 0.015 | 0.037 | 0.068 | 0.880 |
| C9 | 0.045 | 0.099 | 0.133 | 0.724 |
| C10 | 0.066 | 0.077 | 0.112 | 0.745 |
| C11 | 0.091 | 0.128 | 0.110 | 0.671 |
| C12 | 0.109 | 0.130 | 0.156 | 0.605 |

Abbreviations: NMSL, non methylation; HMSL, hemi-methylated proportion; INCML, inner cytosine methylation; FML, full methylated.

**Table S6. Non-hierarchical and hierarchical analysis of molecular variance (AMOVA) of AFLP variation based on groupings of *C. demersum* in Liangzi Lake**

| Source of variation | Df | Sum of squares | Variance components | % variance | ø-statistic^a^ |
| --- | --- | --- | --- | --- | --- |
| Among populations | 11 | 1519.927 | 7.97 | 11.0 | ø_ST_ = 0.108 |
| Within populations | 98 | 6439.219 | 65.71 | 89.0 | NC |
|  |  |  |  |  |  |
| Two genetic groups |  |  |  |  |  |
| Among groups | 1 | 490.595 | 7.03 | 9.1 | ø_CT_ = 0.091 |
| Among populations within groups | 10 | 1080.300 | 4.80 | 6.2 | ø_SC_ = 0.062 |
| Within populations | 98 | 6388.250 | 65.19 | 84.6 | ø_ST_ = 0.154 |
| Total | 109 | 7959.145 | 77.02 |  |  |

Abbreviations: AMOVA, analysis of molecular variance; Df, degrees of freedom; NC, not computed.

^a^All variance components were significant (*P* < 0.001) based on 999 permutations.

**Figure S1.** Estimated genetic structures of *C. demersum* populations in Liangzi Lake. Left, results for *K* = 2-4 clusters (a-c); each individual’s genotype is represented by a vertical line. Populations are separated by black bars and identified at the bottom. Right (d), estimation of an ad hoc quantity (∆*K*) from calculated *K* and LnP (D).


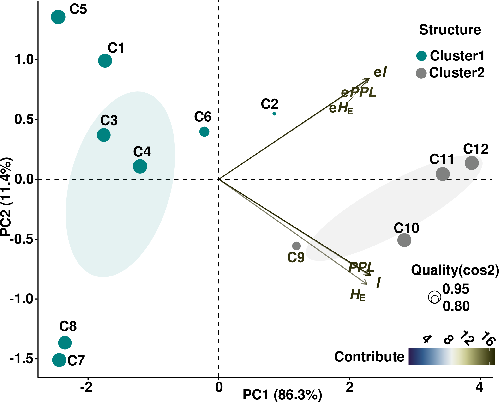


**Figure S2.** PCA scatterplot of genetic structure in *C. demersum* populations. Percentages of explained variation of the two principal axes are annotated. Contributes of genetic and epigenetic variables are represented with scale bar, where the quality of presented variables is showed by circles.

**Figure S3.** Environmental variables associated with genetic and epigenetic variation by correlation and best random forest model in *C. demersum* populations. (a, b) The heatmaps show the estimated importance of relevant environmental variables to genetic (a) and epigenetic (b) indices. (c) The top 8 environmental variables (ranked in descending order of importance to the accuracy of the model) were identified based on the relevance of genetic clusters in *C. demersum* populations. The inset represents ten-fold cross-validation error as a function of the number of input environmental variables used to differentiate two genetic clusters in order of importance of the variables.

**Figure S4.** Procrustes analysis of the correlation of genetic and epigenetic variation with environmental variables according to the NMDS results of environmental variables and genetic clusters (*M*^2^ = 0.514, *P* = 0.005, 999 permutations).

**Figure S5.** Genetic variants outiers associated with the environmental variables. (a) Quantile-Quantile plot for -Log10 *P*-values from association tests. (b) Manhattan plot for genetic variants associated with the Water-body temperature (WT) (brown point),Annual Mean Temperature (AMT) (blue point), Mean Temperature of the Wettest Quarter (MTWeQ) (green point), and Mean Temperature of the Coldest Quarter (MTCoQ) (orange point). The gray dashed horizontal lines represent significance thresholds (Bonferroni correction, adjusted *P* = 0.05). The upper straight lines represent significance thresholds (FDR correction, adjusted *P* = 0.01). Selected candidate locis are labeled in the plot at their respective probe positions.

**Figure S6.** Epigenetic variants outiers associated with the environmental variables. (a) Quantile-Quantile plot for -Log10 *P*-values from association tests. (b) Manhattan plot for epigenetic variants associated with Water-body temperature (WT)( brown point), Annual Mean Temperature (AMT) (blue point), Mean Temperature of the Wettest Quarter (MTWeQ) (green point), and Mean Temperature of the Coldest Quarter (MTCoQ) (orange point). The gray dashed horizontal lines represent significance thresholds (Bonferroni correction, adjusted *P* = 0.05). The upper straight lines represent significance thresholds (FDR correction, adjusted *P* = 0.01). Selected candidate locis are labeled in the plot at their respective probe positions.

**Figure S7.** Multi-Layer Perception (MLP) for classification among *C. demersum* populations. (a) Prediction model with 2×2 hidden layers. Green lines represent positive effects and brown lines represents negative effects. Numbers adjacent to lines are connection weights. (b) Environmental importance of clustering prediction. (c) Confusion matrix of precision of acuracy.

**Supporting-information references:**

Agapow, P.M., Burt, A., 2001. Indices of multilocus linkage disequilibrium. Molecular Ecology Notes 1, 101-102.

Baurens, F.-C., Causse, S., Legavre, T., 2008. Methylation-sensitive amplification polymorphism (MSAP) protocol to assess CpG and CpNpG methylation in the banana genome. Fruits 63, 117-123.

Brown, A., Feldman, M., Nevo, E., 1980. Multilocus structure of natural populations of Hordeum spontaneum. Genetics 96, 523-536.

Simpson, E.H., 1949. Measurement of diversity. Nature 163, 688-688.

Smith, J.M., Smith, N.H., O'Rourke, M., Spratt, B.G., 1993. How clonal are bacteria? Proceedings of the National Academy of Sciences 90, 4384-4388.

Vos, P., Hogers, R., Bleeker, M., Reijans, M., Lee, T.v.d., Hornes, M., Friters, A., Pot, J., Paleman, J., Kuiper, M., 1995. AFLP: a new technique for DNA fingerprinting. Nucleic acids research 23, 4407-4414.
